# Predicting Amputation Rates in Acute Limb Ischemia: Is the Neutrophil-Lymphocyte Ratio a Reliable Indicator?

**DOI:** 10.7759/cureus.59253

**Published:** 2024-04-29

**Authors:** Mehmet Emir Erol, İsa Civelek, Sertan Ozyalcin, Deniz Sarp Beyazpınar, Ozer Kandemir

**Affiliations:** 1 Department of Cardiovascular Surgery, Ankara Etlik City Hospital, Ankara, TUR

**Keywords:** limb ischemia, vascular, neutrophil lymphocyte ratio, amputation, extremity

## Abstract

Objective

This study aimed to investigate the causes of amputation and the associated biochemical parameters in patients with acute limb ischemia (ALI).

Methods

Patients who presented to our clinic with ALI between January 2012 and January 2022 were deemed eligible for participation. Patients who developed ALI owing to atherosclerosis or atrial fibrillation were included in the study. In contrast, patients who developed ALI owing to trauma, iatrogenic causes, or popliteal artery aneurysms were excluded. Patients' demographic data, biochemical parameters, and hemogram values at the time of admission were retrospectively analyzed.

Results

A total of 374 patients were included in the study. Of them, 57.82% (n = 218) were male and 42.18% (n= 156) were female. Amputation was required in 7.95% (n = 30) of the patients after presenting with ALI and receiving necessary surgical or medical intervention. Multivariate analysis revealed the symptom-to-door time to be the primary factor determining the need for amputation in patients. With each passing hour following the manifestation of symptoms, the risk of amputation increased by 1.3 times [odds ratio (OR): 1.289%, 95% confidence interval (CI): 1.079-1.540 p = 0.05]. The neutrophil-to-lymphocyte ratio (NLR) and other hematological parameters had no effect on amputation in both univariate and multivariate analyses (OR: 1.49%; 95% CI: 0.977-2.287 p = 0.512).

Conclusions

Based on our findings, the main factor affecting the need for amputation in ALI patients was the symptom-to-door time. Biochemical and hematological parameters had no effect on amputation in ALI.

## Introduction

Acute limb ischemia (ALI) is a clinical condition that arises from the sudden disruption of blood flow in the arteries of the extremities owing to any etiology [1]. ALI requires urgent intervention and may result in limb loss and mortality if left untreated; in some cases, it represents the last stage of the disease for the patient before they succumb to it [1]. ALI is usually observed in patients with advanced age and a high number of comorbidities. If the symptoms occur <2 weeks before the patient seeks medical attention, it is considered ALI [2]. For the classification of ALI and determination of its clinical course, the Rutherford Classification System (RS) is used, which is based on the assessment of blood flow in the extremities via Doppler ultrasonography and the evaluation of tissue viability [3]. In this classification, Stage 1 does not involve limb loss, whereas patients with Stage 3 disease require amputation.

RS Stage 2a and 2b patients are on the knife’s edge, and limb loss is inevitable in the absence of appropriate interventions. Despite the development of catheter-based endovascular treatments along with embolectomy, which is the most basic procedure in interventional treatment options in these patients, amputation rates in ALI remain high [4]. Approximately 1.5 out of every 10,000 ALI cases undergo major amputations annually [5]. Owing to the potential for both limb loss and mortality, ALI requires rapid and effective implementation of diagnostic methods and appropriate treatment options for the patient. Recently, various studies have been conducted to predict the risk of amputation, mortality, and other adverse effects in patients with ALI, by examining biochemical markers of ischemia and hematological parameters such as the neutrophil-to-lymphocyte ratio (NLR).

In light of this, this study aimed to investigate the factors influencing the need for amputation in ALI patients and to determine whether hematological parameters could be used as predictors of amputation in this patient population.

## Materials and methods

A total of 374 patients who presented to our clinic with ALI and underwent surgical treatment between January 2012 and January 2022 were retrospectively analyzed; the data were collected from the hospital database. We adhered to the principles of the Declaration of Helsinki in conducting this study. The study was approved by the Etlik City Hospital Ethics Committee (26/07/2023- AEŞH-EKI-2023-321). Patients under 18 and over 85 years of age, patients with ALI owing to trauma or popliteal artery aneurysm, Rutherford Stage 1-3 patients, and patients with ALI owing to iatrogenic causes were excluded from the study. All patients included in the study were in Rutherford Stage 2a and Stage 2b.

Demographic data and biochemical and hematological parameters were documented. Data were analyzed by classifying the patients into two groups: 30 patients requiring amputation (Group 1) and 344 patients not requiring amputation (Group 2). The two groups were compared in terms of demographic characteristics, symptom-to-door time, and hematological parameters. Logistic regression analysis was performed to identify risk factors in patients requiring amputation.

Statistical analysis

Continuous variables were presented as mean ± standard deviation (SD) or as median. Categorical variables were presented as numbers and percentages. Comparisons between groups were done using the Pearson chi-square (χ2) test or Fisher’s exact test for categorical variables and the independent samples t-test or Mann-Whitney U test for continuous variables, depending on their distribution. Normality assumptions were tested using the Kolmogorov-Smirnov test. Patients were categorized into two groups according to the presence or absence of amputation [Group 1 (n = 30) and Group 2 (n = 344)]. Differences between the groups were individually calculated in univariate analysis. Variables for which the unadjusted p-value was <0.10 were selected for the multivariable analysis. Odds ratios (OR) with 95% conﬁdence intervals (CI) were calculated using binary logistic regression. The optimal cut-off value for the symptom-to-door time was selected based on the maximum Youden’s index obtained from a receiver operating curve (ROC) analysis to discriminate between patients who were amputated and those who were not.

## Results

A total of 374 patients were analyzed in the study. Of these, 218 were male (58.2%); the mean age of all patients was 69.9 ±12.4 years. Table 1 shows the demographic characteristics and preoperative hematological parameters of the patients. No incidence of mortality was observed throughout the study period.

**Table 1:** Demographic characteristics and preoperative hematological parameters of the patients ^a^t-test; ^b^chi-square test SD: standard deviation; WBC: white blood cell count; MCV: mean corpuscular volume; MCHC: mean corpuscular hemoglobin concentration; PLT: platelet count; PDW: platelet distribution width; NEU: neutrophil count; MCH: mean corpuscular hemoglobin; NLR: neutrophil-lymphocyte ratio; CAD: coronary artery disease; COPD: chronic obstructive pulmonary disease; CVD: cerebrovascular disease; AF: atrial fibrillation

Variables	Amputation (n = 30)	Non-amputation (n = 314)	P-value
Age, years, mean ±SD	70.09 ±11.92	68.13 ±17.38	0.408^a^
Male, n (%)	18 (60)	200 (58)	0.901^b^
Female, n (%)	12 (40)	144 (42)	
Symptom-to-door time, hours, mean ±SD	13.52 ±9.18	33.30 ±9.30	0.001^a^
Hemoglobin, mg/dl, mean ±SD	11.97 ±6.44	12.75 ±3.67	0.145^a^
Hematocrit, mean ±SD	39.06 ±7.24	38.02 ±8.58	0.458^a^
WBC, mean ±SD	11.97 ±6.44	12.75 ±3.67	0.513^a^
MCV, mean ±SD	86.93 ±18.46	88.64 ±6.25	0.614^a^
MCHC, mean ±SD	32.86 ±1.90	32.43 ±1.04	1.04^a^
PLT, mean ±SD	255.07 ±102.67	249.93 ±249.93	0.789^a^
PDW, mean ±SD	13.00 ±2.74	13.57 ±3.40	0.290^a^
NEU, mean ±SD	9.14 ±6.26	9.85 ±6.26	0.536^a^
MCH, mean ±SD	28.23 ±3.45	28.75 ±2.13	0.416^a^
NEU percentage, mean ±SD	73.44 ±12.73	77.45 ±8.76	0.092^a^
NLR, mean ±SD	8.05 ±7.22	9.51 ±12.99	0.508^a^
CAD, n (%)	8 (26)	116 (33)	0.431^b^
Diabetes mellitus, n (%)	19 (63)	44 (12)	0.001^b^
COPD, n (%)	2 (6)	14 (4)	0.500^b^
CVD, n (%)	13 (43)	66 (19)	0.002^b^
AF, n (%)	6 (20)	92 (26)	0.420^b^

When Group 1 and Group 2 were compared, no statistical difference was found between the patients in terms of parameters such as age, sex, hemoglobin, neutrophil count, platelet count, platelet distribution width, or NLR (Table 1). The symptom-to-door time was 13.52 ±9.18 hours in patients who did not require amputation and 33.30 ± 9.30 hours in those who required amputation, and the difference between the two groups was statistically significant (p = 0.001). When comorbid factors were compared, diabetes mellitus (DM) and cerebrovascular disease (CVD) were more common in patients who required amputation (p = 0.001, p = 0.002, respectively), whereas atherosclerotic coronary heart disease, atrial fibrillation (AF), and chronic obstructive pulmonary disease (COPD) were not statistically different between the patient groups (p = 0.431, p = 0.420, and p = 0.5, respectively). Among 374 patients, embolization-related etiology was identified in 98 (26%) cases, while in the remaining patients, the etiology was attributed to in-situ thrombosis.

When patients were evaluated using logistic regression analysis to identify risk factors for amputation, multivariate and univariate regression analysis showed no statistically significant increase in risk concerning age, sex, or any of the hematological parameters. However, symptom-to-door time, DM, and CVD were identified as risk factors for amputation in both multivariate and univariate regression analysis (Table 2). In univariate analysis, preoperative AF was not found to be a singular risk factor. However, in multivariate analysis, it was determined to be a risk factor for amputation (Table 3).

**Table 2:** Univariate regression analysis CI: confidence interval; WBC: white blood cell count; MCV: mean corpuscular volume; MCHC: mean corpuscular hemoglobin concentration; PLT: platelet count; PDW: platelet distribution width; NEU: neutrophil count; MCH: mean corpuscular hemoglobin; NLR: neutrophil-lymphocyte ratio; CAD: coronary artery disease; COPD: chronic obstructive pulmonary disease; CVD: cerebrovascular disease; AF: atrial fibrillation

Variables	Odds ratio (95% CI)	P-value
Sex	0.831 (0.507-2.328)	0.831
Age	0.988 (0.961-1.016)	0.408
WBC	1.018 (0.966-1.073)	0.513
Hemoglobin	0.894 (0.768-1.040)	0.145
Hematocrit	0.981 (0.934-1.031)	0.457
MCV	1.004 (0.988-1.020)	0.620
MCHC	0.896 (0.750-1.070)	0.226
PLT	0.999 (0.996-1.003)	0.788
PDW	1.072 (0.942-1.221)	0.290
NEU	1.017 (0.964-1.074)	0.537
MCH	1.052 (0.932-1.187)	0.414
NEU%	1.028 (0.995-1.062)	0.094
NLR	1.020 (0.962-1.081)	0.507
Symptom-to door time	1.134 (1.096-1.174)	0.001
COPD	0.594 (0.128-2.746)	0.505
Diabetes mellitus	0.85 (0.038-0.195)	0.001
CVD	0.310 (0.144-671)	0.003
AF	1.460 (0.579-3.686)	0.423

**Table 3:** Multivariate regression analysis CI: confidence interval; WBC: white blood cell count; MCV: mean corpuscular volume; MCHC: mean corpuscular hemoglobin concentration; PLT: platelet count; PDW: platelet distribution width; NEU: neutrophil count; MCH: mean corpuscular hemoglobin; NLR: neutrophil-lymphocyte ratio; CAD: coronary artery disease; COPD: chronic obstructive pulmonary disease; CVD: cerebrovascular disease; AF: atrial fibrillation

Variables	Odds ratio (95% CI)	P-value
Sex	3.678 (0.85-159.614)	0.498
Age	0.981 (0.848-1.135)	0.793
WBC	16.606 (0.892-309.295)	0.060
Hemoglobin	0.00 (0-3.372)	0.071
Hematocrit	109.052 (0.665-17887.3)	0.071
MCV	1.960 (0.001-7239.44)	0.872
MCHC	665.007 (0-3.005)	0.604
PLT	1.019 (0.005-1.044)	0.115
PDW	3.046 (0.997-9.309)	0.051
NEU	0.027 (0-1.566)	0.081
MCH	0.144 (0-1.474)	0.881
NEU%	1.236 (0.781-1.955)	0.366
NLR	1.495 (0.977-2.287)	0.512
Symptom-to door time	1.289 (1.079-1.540)	0.005
COPD	0.805 (0.069-9.453)	0.863
Diabetes mellitus	0.119 (0.038-0.373)	0.001
CVD	0.354 (0.116-1.081)	0.05
AF	18.626 (3.632-95.519)	0.001

**Figure 1:**
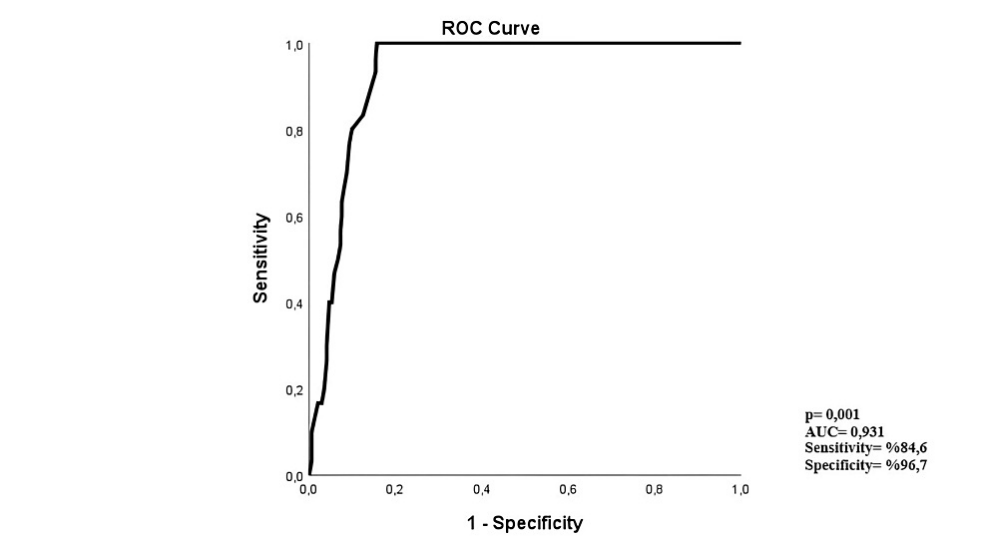
Cut-off value of symptom-to-door time for predicting the need for amputation ROC: receiver operating characteristic curve; AUC: area under the ROC curve

ROC curve analysis was performed to determine the cut-off value of symptom-to-door time for predicting the need for amputation (Figure 1). In this analysis, the cut-off symptom-to-door time for amputation was 19.5 hours (p = 0.001, AUC: 0.931, sensitivity: 96.7%, specificity: 84.6%).

## Discussion

The main finding of this study was that hematological parameters had no effect on amputation rates in Rutherford Stage 2a and Stage 2b patients with ALI. Instead, the most important factor affecting amputation in ALI patients was the symptom-to-door time, i.e., the time from the onset of symptoms until the patient presented to the hospital.

Especially in recent years, researchers have examined numerous hematologic and biochemical parameters to determine mortality and risk of amputation in patients who develop ALI. According to the guidelines of the European Society for Vascular Surgery (ESVS), there is no biochemical parameter to predict limb salvage or determine mortality in cases of ALI [2]. NLR is undoubtedly the most studied parameter. NLR is obtained from the ratio of neutrophil values to lymphocyte values in a simple hemogram test and is an indicator of the adaptive response of the immune system, which is mainly provided by neutrophils and supported by lymphocytes [6]. Elevated NLR values can be seen in several conditions, including bacterial or fungal infections [7-8], acute stroke [9], myocardial infarction [10], severe trauma [11], and cancer [12]. In ALI, most researchers have argued that NLR is a biochemical marker of amputation and early morbidity and mortality. In a study by Pasqui et al., it was found that preoperative NLR could provide a prognostic evaluation of clinical outcomes in RS Stage 2 patients with a cut-off value of 5 [13]. In the same study, the researchers stated that the true predictive value of their results had statistical significance.

In another recent study, by Coelho et al., high preoperative NLR values were observed to be associated with mortality and amputation in the first 30 days [14]. However, one of the limitations of that study, noted by the researchers themselves, was that the blood samples of the patients were not taken homogeneously during the study, which might have affected the results. In the context of the same study, extended ischemic duration was deduced as a contributing factor influencing both mortality and likelihood of amputation. In the present study, NLR had no statistically significant effect on amputation rates. The ischemic period was identified as the main factor affecting the need for amputation after ALI.

In light of these findings, two major issues arise: firstly, is NLR, which tends to increase in any inflammatory condition, actually increased owing to ALI in patients presenting with ALI and multiple comorbidities or is it high due to the inflammatory process caused by other comorbid characteristics? According to our hypothesis and the results of this study, NLR has no predictive value on the need for amputation in patients presenting with ALI. Univariate and multivariate analyses supported our conclusion. The second issue pertains to the fact that most studies on NLR and other hematological and biochemical parameters, to the best of our knowledge, have not adequately considered the time factor, which is the essential factor affecting the need for amputation in ALI. If NLR is elevated in inflammatory or ischemic processes, it would be quite reasonable for it to be elevated in patients with a higher ischemic burden, such as those with ALI. In Coelho’s study, patients requiring amputation were older, had more comorbidities, and had longer ischemic durations. These findings also support our hypothesis. Furthermore, the term “predictor” is occasionally misused to describe associated conditions or situations [15], which may affect the relationship between biochemical parameters and clinical status. Hence, it is important to build a robust model involving an improved variable selection process and at least sample validation [16]. Furthermore, our study demonstrated that multisystem vascular diseases such as DM and CVD further increase the risk of amputation in peripheral vascular disease, as expected.

Finally, despite all these debates, ALI remains one of the most critical emergency clinical conditions in vascular surgery, characterized by high mortality and morbidity, necessitating urgent intervention. Undoubtedly, identifying biochemical or hematological parameters capable of predicting the patient’s prognosis in this emergency, beyond clinical examination and imaging methods, is one of the greatest hopes for vascular surgeons. Unfortunately, as mentioned in this study, such a laboratory parameter has yet to be identified. As researchers, we believe that the most critical factors affecting the clinical prognosis of ALI are the patient’s comorbidities and the time elapsed from the onset of symptoms to hospital admission. The results obtained from this study support this view.

The most important limitations of this study are its single-center design and retrospective nature. In addition, blood samples might not have been collected and analyzed at the same time in all patients included in the study. Lastly, the effect of NLR on mortality could not be analyzed because there were no deceased patients in the study.

## Conclusions

Our findings indicate that in ALI cases, the rates of amputation predominantly hinge on patients' comorbidities and the duration between symptom onset and hospital admission, surpassing the influence of hematologic parameters. However, we believe that further comprehensive studies are necessary to fully comprehend the significance of NLR or any other more specific parameters in ALI.

## References

[1] Sidawy AN, Perler BA (2022). Rutherford's Vascular Surgery and Endovascular Therapy. Sidawy,MD,MPH and Bruce A Perler,MD,MBA. Rutherford’s Vascular Surgery and Endovascular Therapy.

[2] Björck M, Earnshaw JJ, Acosta S (2020). Editor's Choice - European Society for Vascular Surgery (ESVS) 2020 clinical practice guidelines on the management of acute limb ischaemia. Eur J Vasc Endovasc Surg.

[3] Rutherford RB, Baker JD, Ernst C, Johnston KW, Porter JM, Ahn S, Jones DN (1997). Recommended standards for reports dealing with lower extremity ischemia: revised version. J Vasc Surg.

[4] de Donato G, Pasqui E, Sponza M (2021). Safety and efficacy of vacuum assisted thrombo-aspiration in patients with acute lower limb ischaemia: The INDIAN Trial. Eur J Vasc Endovasc Surg.

[5] Byrne RM, Taha AG, Avgerinos E, Marone LK, Makaroun MS, Chaer RA (2014). Contemporary outcomes of endovascular interventions for acute limb ischemia. J Vasc Surg.

[6] Buonacera A, Stancanelli B, Colaci M, Malatino L (2022). Neutrophil to lymphocyte ratio: an emerging marker of the relationships between the immune system and diseases. Int J Mol Sci.

[7] Lowsby R, Gomes C, Jarman I (2015). Neutrophil to lymphocyte count ratio as an early indicator of blood stream infection in the emergency department. Emerg Med J.

[8] Jiang J, Liu R, Yu X, Yang R, Xu H, Mao Z, Wang Y (2019). The neutrophil-lymphocyte count ratio as a diagnostic marker for bacteraemia: a systematic review and meta-analysis. Am J Emerg Med.

[9] Li W, Hou M, Ding Z, Liu X, Shao Y, Li X (2021). Prognostic value of neutrophil-to-lymphocyte ratio in stroke: a systematic review and meta-analysis. Front Neurol.

[10] Lee MJ, Park SD, Kwon SW (2016). Relation between neutrophil-to-lymphocyte ratio and index of microcirculatory resistance in patients with st-segment elevation myocardial infarction undergoing primary percutaneous coronary intervention. Am J Cardiol.

[11] Park JM (2017). Neutrophil-to-lymphocyte ratio in trauma patients. J Trauma Acute Care Surg.

[12] Lee PY, Oen KQ, Lim GR (2021). Neutrophil-to-lymphocyte ratio predicts development of immune-related adverse events and outcomes from immune checkpoint blockade: a case-control study. Cancers (Basel).

[13] Pasqui E, de Donato G, Giannace G (2022). The relation between neutrophil/lymphocyte and platelet/lymphocyte ratios with mortality and limb amputation after acute limb ischaemia. Vascular.

[14] Coelho NH, Coelho A, Augusto R (2021). Pre-operative neutrophil to lymphocyte ratio is associated with 30 day death or amputation after revascularization for acute limb ischemia. Eur J Vasc Endovasc Surg.

[15] Varga TV, Niss K, Estampador AC, Collin CB, Moseley PL (2020). Association is not prediction: a landscape of confused reporting in diabetes - a systematic review. Diabetes Res Clin Pract.

[16] Meuli L, Zimmermann A (2021). Neutrophil to lymphocyte ratio: a long way from association to prediction. Eur J Vasc Endovasc Surg.

